# Violence Experience among Immigrants and Refugees: A Cross-Sectional Study in Italy

**DOI:** 10.1155/2018/7949483

**Published:** 2018-09-23

**Authors:** Francesco Napolitano, Luciano Gualdieri, Gabriella Santagati, Italo Francesco Angelillo

**Affiliations:** ^1^Department of Experimental Medicine, University of Campania “Luigi Vanvitelli”, Via Luciano Armanni, 5 80138 Naples, Italy; ^2^Hospital “Cardinale Ascalesi”, Local Health Unit Naples 1, Via Egiziaca a Forcella, 31 80139 Naples, Italy

## Abstract

The objectives of this cross-sectional investigation among a random sample of immigrants and refugees in Italy were to gain an insight into the extent and type of the episodes of violence and to assess their association with different characteristics. Data was collected from September 2016 to July 2017 using a face-to-face structured interview. A total of 503 subjects participated. Overall, 46.5% and 40% of the sample reported having experienced some form of violence in Italy at least once since they arrived and during the last 12 months. Psychological violence was the most common form experienced by 53.2% of the participants, 40.3% experiencing physical violence, 18.9% economic violence, and only 6.5% intimate partner violence. The risk of experiencing at least one form of violence in the last 12 months in Italy was more likely to occur among immigrants who have been in Italy much longer and less likely in those who lived in a camp. The number of episodes of violence experienced since they arrived in Italy was significantly higher in female, in those who have been in Italy much longer and in those who had experienced at least one racially discriminatory episode of violence, whereas those with middle and high school or above educational level and those who did not experience psychological consequences of the violence had experienced a lower number of episodes. These results must be used to strengthen interventions and policies aimed at preventing violence among this population.

## 1. Introduction

Immigration is a worldwide phenomenon that is constantly increasing over the last few years and, in 2015, approximately 18 million of immigrants, both documented and undocumented, asylum seekers, and refugees, entered a new country [1]. Italy is among the countries with the highest number of migrants and in 2016 as more than 240.000 people arrived, with most being from North and sub-Saharan Africa, either in search of better economic and social opportunities or having been forced to flee crises and political conflicts [2]. In particular, in 2017 approximately 240.000 immigrants lived in the Campania Region and more than 120.000 in the geographical area of Naples [3].

After arriving in their new country, immigrants face many difficulties, for example, social isolation, unemployment, poverty, language difficulties, cultural background, and stressful living conditions, and these factors have been suggested as possible determinants of immigrants' health. Therefore, this makes them a particularly vulnerable population and although efforts have been made in promoting health equity, being an immigrant is associated with a lower use of the different levels of healthcare and a greater risk for several negative health-related outcomes such as, for example, obesity [4–6], discrimination or violence [7, 8], and mental health disorders [9, 10].

In Southern Italy previously published data have focused their attention on investigating the health conditions, mainly the epidemiological characteristics of different diseases [11–13] and the patterns of access and utilization of healthcare services [14] by groups of immigrants. To date, there is relatively little work examining the occurrence of violence in these communities [7, 15–18], despite the fact that this is a significant global public health problem. Hence, the urgency to better understand the prevalence of the violence experienced within the immigrant groups is crucial in order to implement appropriate public health strategies. Therefore, the primary objective of the current investigation was to gain an insight into the extent and type of the episodes of violence among a large group of immigrants and refugees in Italy and then to assess their association with different characteristics.

## 2. Materials and Methods

### 2.1. Study Design and Setting

The study has a cross-sectional observational design. Data collection started at the beginning of September 2016 and ended in July 2017 in an Ambulatory Center of the “Cardinale Ascalesi” Public General Hospital in Naples, Italy, that provides medical care to 3000 immigrants every year who are temporarily present in Italy and not in compliance with the entry regulations, regular immigrants, refugees, or asylum seekers.

### 2.2. Participants and Recruitment

A random sample of 587 immigrants and refugees who attended the Ambulatory Center for a health assessment was selected. In order to determine the number of subjects needed, the sample size was obtained using the formula for cross-sectional study designs with the following assumptions: proportion that 30% of immigrants were victims of violence, a confidence interval of 95%, an error of 5%, and a response rate of 80%. Therefore, the minimum required sample size was determined in 400 participants.

Two medical researchers, unaffiliated with the Ambulatory Center staff and well trained in data collection, approached the potential participants in the waiting room of the Ambulatory Center and invited them to take part in the survey. The participants were given oral information about the objectives of the study, that their participation was voluntary, that they could withdraw from the study at any time without explanation, that the answers were to be given anonymously, that no personal identification data would be recorded, and that all information would be kept confidential. Voluntary signed informed consent was obtained from every participant before beginning each interview. No incentive or reimbursement was offered to the respondents.

### 2.3. Instrument

A structured questionnaire was completed during a face-to-face interview with the two trained researchers and when necessary with the assistance of a cultural mediator, routinely present in the Ambulatory Center, as a translator if the subject did not speak Italian. Each interview took approximately 20 minutes to be completed and it was conducted in a private and quiet place during the waiting time. The research team developed the questionnaire based on interviews with healthcare providers, relevant literature, and previous experiences. The questionnaire consisted of 20 items divided into four sections. The first section included questions on participants' sociodemographic and general characteristics. In the second section respondents were asked to report the number of their experience as victims of episodes of violence in Italy at any time since their arrival and in the 12 months prior to the survey. Respondents who reported at least one episode of violence were asked to describe the last three experiences.

Specifically, participants were classified as having experienced violence if they reported any of the following acts: physical violence: being slapped, pushed, hit, kicked, dragged or beaten up, choked or burnt, and threatened or hurt with an object; sexual violence: being physically forced to have sexual intercourse, having had unwanted sexual intercourse because of fear of what the partner might do, and being forced to perform other sexual acts that the respondent found degrading or humiliating; psychological violence: being yelled at, insulted, humiliated, made to feel inadequate, threatened, and in fear or put in danger of being belittled or made to feel bad about him/herself and having someone doing things to scare or intimidate him/her on purpose; economic violence: being stopped or having someone trying to stop them from having access to household money, being stopped or having someone trying to stop them from working or earning money, and being deprived of the basic needs. 

To determine the level of understanding of the questions contained in the questionnaire, a pilot study was conducted among 25 subjects, not included in the final sample, and revised before being finalized.

The Ethics Committee at the Teaching Hospital of the University of Campania “Luigi Vanvitelli” approved the study protocol.

### 2.4. Data Analysis

Statistical analyses were conducted in several steps. First, descriptive analyses including frequency distribution, mean, median, standard deviation, and range have been used to characterize the participating subjects. Next, independent *t* for continuous variables or chi-square tests for binary variables were carried out to investigate the bivariate relationship between independent and dependent variables. Candidate variables were included in the corresponding multivariable models if their* p*-value was less than or equal to 0.25 in the univariate analysis. As a final step, multivariable ordered and logistic regression models, respectively, for ordinal and dichotomous outcomes, were constructed to examine the effects of major independent variables on the following outcomes of interest: (a) profile of the subject who has experienced at least one form of violence in the last 12 months in Italy (Model 1); (b) profile of the subject according to the number of episodes of any form of violence experienced since arriving in Italy (1=1; 2-5=2; >5=3) (Model 2). The following independent variables were included in all models: age (continuous, in years), gender (male=0; female=1), regional origin (Africa=1; Eastern Europe=2; Asia/South America=3), educational level (illiterate=1; primary school=2; middle school=3; high school and above=4), religion (Atheist=1; Christian/Catholic=2; Muslim=3; other=4), length of stay in Italy (continuous, in months), employment status (unemployed=0; employed=1), and living conditions (house=1; camp=2; street=3). Moreover, the variables types of violence (psychological=1; physical/sexual=2; economic=3), perpetrator of the violence (unknown=0; known =1), site of the violence (street/public transport=0; working place/home/camp=1), experiencing at least one racially discriminatory episode of violence (no=0; yes=1), experiencing psychological consequences of the violence (no=0; yes=1), and reaction to the violence (no=0; yes=1) were included in Model 2. Stepwise multivariate regression with an inclusion and exclusion level respectively of 0.2 and 0.4 was used to determine the final models. Multivariate regression models presented odds ratios (ORs) and 95% confidence intervals (CIs) to reflect association strength between the different variables and the outcomes of interest. All statistical tests were two-tailed, and a* p*-value below or equal to 0.05 was used to determine statistical significance. Stata software version 15 was used to perform all statistical analyses of the data [19].

## 3. Results

Of the 587 immigrants and refugees randomly selected for participation in the study, the final sample included 503 subjects for an overall response rate of 85.7%.

Overviews of the principal characteristics concerning the participants are presented in detail in Table 1. In brief, the average age was 35.3 years (range from 15 to 74), more than half were male and originated from countries in sub-Saharan Africa, 13.7% had no formal education, almost half was single, one-third was with families, almost half was Muslim, the majority came because of economic reasons, two-thirds lived in a house, one-third had at least one family member living with them in the same house, more than half was currently unemployed, the median time of length of stay in Italy was 4 years, and 28.6% had arrived less than one year ago.

Table 2 shows the characteristics of the self-reported episodes of the different form of violence that had occurred in Italy at some point from their arrival and in the last 12 months in the study population. Of the study sample, 46.5% and 40% reported having experienced some form of violence at least once since they arrived in Italy and in the last 12 months, with a median of 2 and 1 different victimization forms, respectively. A total of 21.1% had been victims of more than one form of violence and almost one out of four subjects experienced four types of violence at some time during their stay in Italy. In particular among women, 56% had been victims of at least one form of violence during their stay in Italy and 28.5% experienced multiple victimizations. Among those who suffered one episode, 85.9% claimed to have been victims of violence during the 12 months prior to the survey. Psychological violence was the most common form experienced by more than half (53.2%) of the participants, 40.3% had experienced physical violence, 18.9% had experienced economic violence, and only 6.5% had intimate partner violence. More than half (61.7%) reported that they have experienced at least one episode of violence with racial discrimination. Among those who were employed, 58.1% reported having been experienced economic violence, and among those who had a partner 53.8% reported an intimate violence.

The perpetrators were unknown to the victim in more than two-thirds of the episodes (68.6%), with the exception of those perpetrated by the employer (20.9%) and by partners (6.5%). With regards to the site of the violence, more than two-thirds (68.6%) took place in the street, while 21.9% and 10.4% occurred in the workplace and at the victim's home, respectively. The immediate main health consequence was a contusion in more than half of the episodes (54.3%), followed by cutting wounds (17.3%), fractures (16%), and lacerations (14.8%). Moreover, more than half reported stress and anxiety disorders (e.g., posttraumatic stress disorder) and approximately one-third reported a chronic anxiety. In three-quarters of the episodes, the victim did not tell anyone about the violence. More than a quarter (27.4%) considered very seriously these experiences of violence, with an overall mean value of 7.4 out of a maximum score of 10.

Results from the bivariate analysis showed that having experienced at least one form of violence (psychological, physical, economic, and sexual) in the last 12 months in Italy was significantly more likely among females (*χ*^2^=* *5.03;* p*=0.02), the employed (*χ*^2^=3.84;* p*=0.03), those who lived in a house (*χ*^2^=* *40.72;* p*<0.0001), and those with a longer length of stay in Italy (t=* *-5.36;* p*<0.0001).  Table 3 shows the adjusted ORs and 95% CIs of the final multivariable stepwise logistic and ordered regression models performed to explore the associations between the various characteristics and the different outcomes of interest. After adjustment for the several characteristics, the results partially confirmed those of the univariate analysis and the variables of living conditions and length of stay in Italy resulted significantly and independently associated with experiencing at least once some form of violence in the last 12 months in Italy. Indeed, there is clear evidence that the risk of experiencing at least one form of violence in the last 12 months in Italy was more likely to occur among those who have been in Italy much longer (OR=1.01; 95% CI: 1.003-1.01) and less likely in immigrants who lived in a camp (OR=0.29; 95% CI: 0.17-0.51) compared to those who lived in a house. Females have 41% greater chance of experiencing a form of violence than males although the difference was not statistically significant (Model 1). In the ordinal logistic regression analysis model, the number of episodes of violence experienced by the participants since they arrived in Italy was significantly higher in females (OR=3.32; 95% CI: 1.65-6.68), in those who have been in Italy much longer (OR=1.005; 95% CI: 1.001-1.01), and in those who had experienced at least one racially discriminatory episode of violence (OR=2.87; 95% CI: 1.54-5.34). Moreover, those with middle school (OR=0.31; 95% CI: 0.12-0.83) and high school or above (OR=0.31; 95% CI: 0.12-0.79) educational level compared to those who were illiterate and those who did not experience in psychological consequences of the violence (OR=0.19; 95% CI: 0.08-0.46) had experienced a lower number of episodes of violence (Model 2).

## 4. Discussion

To our knowledge, this is the first comprehensive assessment that has provided a unique opportunity to evaluate the extent and type of the self-reported episodes of violence within a large group of immigrants and refugees in Italy. Such information is useful in order to promote and implement strategies for reducing forms of violence among this vulnerable population. Indeed, the violence is very important issue that has been investigated among different groups worldwide [20–23].

Formal comparability of the present findings with observations from previous similar international studies is limited by the heterogeneity of the methodology, including differences in the sampling strategies, the population examined, instrument for collection of data, and definition of the forms of violence, since all of which may influence the observed prevalence estimates of violence. According to the data in this study, at least one episode of violence (physical, sexual, psychological, and economic) during their stay in Italy and in the last 12 months was experienced by 46.5% and 40% of the sample of immigrants and refugees, respectively. According to the literature, the prevalence of any form of violence among this population shows remarkable differences worldwide. Namely, the value was considerably lower than the 85.7% reported in sub-Saharan migrants in Morocco or at its borders [15] and the 52.1% in female urban refugees and asylum seekers from the Democratic Republic of Congo and Somalia in Uganda [24]. The value was considerably higher than those described in previous studies conducted across other countries. Indeed, there were 4.1% victimization among immigrants in the US [25], 4.6% and 7.7% of Syrian refugees in their Greek settlement [26], and 6.5% and 12% of Latin American and Moroccan origin in Spain [7], and 15.1% of immigrants in Portugal were victims of at least one episode of violence, regardless of its type, during the past year [18], and there were 15.6%, 10.9%, and 8.6% of intimate partner violence prevalence among Ecuadorian, Moroccan, and Romanian immigrant women living in Spain [17], while 21.1% from Central America and Mexico experienced violence during their transit through Mexico towards the USA [16] and 23.7% and 39% of asylum seekers, refugees, and undocumented migrants, respectively, in eight European countries [27] and in Belgium and the Netherlands [28].

The results of this study are in line with those of the previous publications on these population-based samples showing that the psychological violence was the form experienced more frequently, with more than half of those interviewed having reported an experience of this form of violence in the past 12 months in Italy [29, 30]. In concert with past research, an unknown person was found to be the most predominant offender among those victimized [15].

In an attempt to elucidate independent predictors of the outcomes of interest, the multivariate results revealed, after adjusting for all the selected characteristics, that several factors were uniquely related to the episodes of violence. An important finding seen was that participants who experienced at least one form of violence in the last 12 months in Italy were significantly more likely to be living in house and with a higher number of years in Italy. The risk associated with increasing duration of stay in Italy is in accordance with the already mentioned study of immigrants in Portugal [18]. This finding indicates that the influence of duration of stay on violence will be important to understand and to develop strategies to maintain the immigrant health advantage over time after migration. This remains to be studied. Furthermore, this study supports the observation that violence against women is still a common public health issue worldwide with more than half experiencing some form of physical or sexual violence during their stay in Italy and 28.5% experiencing multiple victimizations. Indeed, global estimates indicate that about 35% women have experienced either physical and/or sexual intimate partner violence or nonpartner sexual violence in their lifetime [31]. The findings regarding the higher frequency of violence in women compared to male was observed in previous studies [16, 18, 29, 32]. This disturbing finding suggests that there is plenty of room for action that should be activated as soon as possible, tailored for reducing this value.

It is important to acknowledge that some limitations of the present study need to be kept in mind when interpreting the findings. First, since this has a cross-sectional design, the study, therefore, precludes assumptions about the causal inferences or the directionality of the associations between the predictor variables and the outcomes of interest. Second, this investigation was performed at one center and, therefore, the results cannot be generalized to the total population of immigrants and refugees in Italy. Third, the questionnaire was based on self-reported responses, which may have predisposed study findings to recall bias and participants do not necessarily remember all the episodes of violence they had during their stay in Italy or in the last 12 months. Therefore, the reported violence may differ from the actual experience suggesting that the true prevalence is more likely to be underreported. The potential for such bias exists in almost all surveys that use self-reported information and when no corroborative objective information is available. Fourth, because of the sensitive nature of the subject matter and varying cultural norms, respondents may avoid sharing such information on violence, especially those of a sexual nature, with a possible underestimate of the true prevalence reported here, though respondents were informed that surveys were confidential and efforts were made in order to make them comfortable during the interview, with the assistance of a cultural mediator familiar with the immigrants, so they could speak freely. Despite the aforementioned limitations, there are several advantages, including the fact that such a study was conducted for the first time in Italy. This investigation, with a large sample size of a difficult to reach population and a very high study participation rate, provides solid results and allows reasonable conclusions to be drawn about the prevalence of the episodes of violence among immigrants and refugees in Italy.

## 5. Conclusions

The results of this study add to the scarce literature on violence within immigrants and refugees and they must be used to strengthen interventions and policies aimed at preventing violence from occurring among this population.

## Figures and Tables

**Table 1:** Sociodemographic characteristics of the study population.

	N	%
Gender		
Male	271	53.9
Female	232	46.1
Age, mean ± SD (range), *years*	35.3±12.7 (15-74)	
Regional origin		
Sub-Saharan Africa	288	57.3
Eastern Europe	101	20.1
Asia	61	12.1
North Africa	28	5.5
South America	25	5
Religion		
Muslim	230	45.7
Catholic	130	25.9
Christian Orthodox	98	19.5
Other	31	6.1
Atheist	14	2.8
Educational level, mean ± SD (range), *years*	10.3±4.5 (1-23)	
Illiterate	69	13.7
Primary school	72	14.3
Middle school	141	28
High school and above	221	44
Marital status		
Single	233	46.3
Married	201	40
Separated/Divorced/Widowed	69	13.7
Number of children, median (range)	2 (1-8)	
0	225	44.7
1	109	21.7
≥2	169	33.6
Living conditions		
House	332	66
Camp	146	29
Street	25	5
Number of cohabiting, median (range)	2 (1-8)	
Length of stay in Italy, median (range), *years*	4 (1 month-37 years)	
Employment status		
Unemployed	282	56.1
Employed	221	43.9

**Table 2:** Frequency and characteristics of the different types of victimization that occurred in the study population.

	**N**	**%**
Experienced at least one episode of violence during their stay in Italy	234	46.5
Experienced at least one episode of violence in the last 12 months in Italy	201	40
Experienced at least one episode of violence with racial discrimination ^a^	124	61.7
Type of violence ^a^		
Psychological	107	53.2
Physical	81	40.3
Economic	38	18.9
Sexual	4	2
Site of the violence ^a^		
Street	138	68.6
Working place	44	21.9
Home	21	10.4
Public transport	20	9.9
Camp	6	3
Perpetrator ^a^		
Unknown people	138	68.6
Employer	42	20.9
Another immigrant	20	9.9
Husband/partner	13	6.5
Acquaintance	9	4.5
Relatives	2	1
Consequences of physical violence ^b^		
Contusions	44	54.3
Cutting wounds	14	17.3
Fractures	13	16
Lacerations	12	14.8
Psychological consequences of the violence ^a^		
Post-traumatic stress disorder	107	53.2
Chronic anxiety	62	30.8
Aggressiveness	19	9.4
Phobias	18	8.9
Sleep disorders	9	4.5
Anger	3	1.5
Depression	2	1
Reaction to the violence ^a^		
None	157	78.1
Go to the hospital	16	7.9
Ask for help from friends/relatives	11	5.5
Go to the police	10	5
Self-medication	7	3.5
Go to a doctor	5	2.5

^a^ Only for those who reported that they had experienced at least one episode of violence in the last 12 months in Italy.

^b^ Only for those who reported that they had experienced at least one episode of physical violence in the last 12 months in Italy.

**Table 3:** Multivariable logistic and ordered regression models indicating associations between several variables and the different outcomes of interest.

**Variable**	**OR**	**SE**	**95% CI**	***p* value**
**Model 1.** Profile of the subject who has experienced at least one form of violence in the last 12 months in Italy				
Log likelihood=-306.86, *χ*^2^=63.16 (7 df), *p*<0.0001				
Length of stay in Italy	1.01	0.01	1.003-1.01	<0.0001
Living condition				
House	1*∗*			
Camp	0.29	0.08	0.17-0.51	<0.0001
Street	1.93	0.79	0.86-4.31	0.11
Female	1.41	0.29	0.93-2.14	0.1
Age	0.99	0.01	0.97-1.01	0.19
Regional origin				
Sub-Saharan Africa	1*∗*			
Eastern Europe	0.71	0.19	0.43-1.19	0.2
Employment status	0.81	0.18	0.53-1.24	0.34

**Model 2.** Profile of the subject according to the number of episodes of any form of violence experienced since arriving in Italy				
Log likelihood=-209.68, *χ*^2^=65.18 (21 df), *p*<0.0001				
Experienced psychological consequences of the violence	0.19	0.08	0.08-0.46	<0.0001
Female	3.32	1.18	1.65-6.68	<0.0001
Experienced at least one racially discriminatory episode of violence	2.87	0.91	1.54-5.34	0.001
Length of stay in Italy	1.005	0.01	1.001-1.01	0.017
Educational level				
Illiterate	1*∗*			
High school and above	0.31	0.15	0.12-0.79	0.015
Middle school	0.31	0.16	0.12-0.83	0.02
Primary school	0.79	0.44	0.26-2.34	0.7
Living condition				
House	1*∗*			
Camp	0.46	0.21	0.19-1.11	0.08
Street	0.41	0.22	0.15-1.16	0.09
Known perpetrator	1.9	0.82	0.81-4.47	0.14
Religion				
Atheist	1*∗*			
Muslim	3.51	3.05	0.63-19.3	0.15
Christian/Catholic	1.34	1.12	0.26-6.99	0.73
Other	1.12	1.01	0.19-6.51	0.9
Type of violence				
Psychological	1*∗*			
Economic	0.55	0.27	0.21-1.43	0.22
Physical/Sexual	0.92	0.29	0.49-1.69	0.79
Age	0.98	0.01	0.96-1.01	0.28
Violence in street and public transport	0.69	0.29	0.3-1.57	0.38
Reaction to the violence	1.36	0.54	0.63-2.95	0.43
Regional origin				
Sub-Saharan Africa	1*∗*			
Eastern Europe	1.31	0.62	0.52-3.32	0.56
Asia/ South America	0.9	0.36	0.41-1.98	0.79
Employment status	1.06	0.32	0.59-1.91	0.84

*∗*Reference category.

## Data Availability

The data used to support the findings of this study are available from the corresponding author upon request.
